# Platelets During Myelin Repair in Multiple Sclerosis: Friend or Foe?

**DOI:** 10.1111/jnc.70268

**Published:** 2025-11-03

**Authors:** Francisco J. Rivera, Amber R. Philp, Carolina R. Reyes, Carlos Valenzuela‐Krugmann, Maria Elena Silva

**Affiliations:** ^1^ Translational Regenerative Neurobiology Group (TReN), Molecular and Integrative Biosciences Research Program (MIBS), Faculty of Biological and Environmental Sciences University of Helsinki Helsinki Finland; ^2^ Department of Anatomy University of California San Francisco San Francisco California USA

**Keywords:** multiple sclerosis, myelin, oligodendrocyte progenitor cells, platelets, remyelination

## Abstract

Multiple sclerosis (MS) is an autoimmune neuroinflammatory demyelinating disease of the central nervous system (CNS) that affects more than 2.5 million people worldwide. Remyelination represents a robust regenerative response to myelin damage; however, during the later stages of MS, this process largely fails. Upon demyelination, oligodendrocyte progenitor cells (OPCs) proliferate, migrate, and differentiate into mature remyelinating oligodendrocytes. Why does remyelination fail in MS? Platelets are small, oval, anucleate cells that circulate in the bloodstream and form a hemostatic plug to stop blood leakage upon endothelial damage. Platelet function is not restricted to hemostasis; they also display tissue‐regenerative activities. Here, we review evidence suggesting that platelets act as modulators of OPC function during remyelination. Additionally, we describe platelet alterations associated with MS that may contribute to remyelination failure. Finally, we highlighted our previous study that addressed these issues. This study showed that in response to myelin damage, platelets transiently accumulate within the lesion. Interestingly, platelet depletion leads to a reduction in OPC differentiation, hindering remyelination. In vitro studies revealed that transient exposure to platelets boosts OPC differentiation, whereas sustained exposure to platelets suppresses this beneficial effect. Consistent with this observation, in an in vivo model of thrombocytosis (*Calr^+/−^*), we found a sustained increase in the number of blood‐borne platelets recruited into the CNS (as observed in MS lesions), resulting in a significant decline in OPC differentiation during remyelination. These findings reveal a complex role of platelets in remyelination and provide new insights for understanding the MS pathology as well as for designing regenerative strategies for the treatment of this disease.

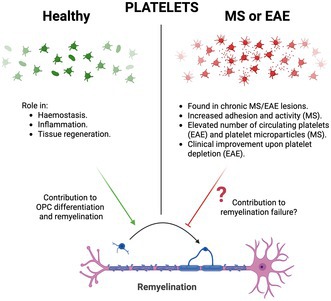

## Graphical abstract

*Platelets support remyelination, while alterations in their function in multiple sclerosis (MS) and experimental autoimmune encephalomyelitis (EAE) may contribute to remyelination failure.* In healthy individuals, platelet function encompasses roles in hemostasis, inflammation, and tissue regeneration, among others. Recent findings indicate that platelets play a crucial role in oligodendrocyte progenitor cell (OPC) differentiation and remyelination. However, in the context of MS and EAE, platelets aggregate in chronic non‐remyelinated lesions, become hyperactive, and exhibit increased adhesiveness. MS patients show an increase in circulating platelet microparticles, while EAE animal models display elevated circulating platelet numbers. These observations, along with recent findings, suggest that alterations in platelet function may contribute to remyelination failure in MS.

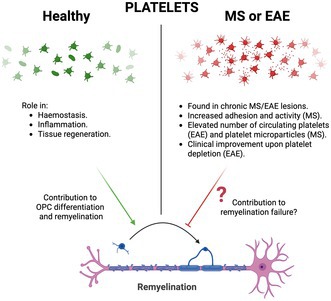

**Abbreviations**
BBB: blood–brain barrier
bFGF: basic fibroblast growth factor
*Calr*
: calreticulin
CNS: central nervous system
dpi: days post‐induction
dpl: days post‐lesion
EAE: experimental autoimmune encephalomyelitis
EGF: epidermal growth factor
FGF‐2: fibroblast growth factor 2
HGF: hepatocyte growth factor
LPC: lysolecithin
MS: multiple sclerosis
NSPC: neural/stem progenitor cell
OPC: oligodendrocyte progenitor cell
PDGF‐AA: platelet‐derived growth factor AA
PNS: peripheral nervous system
VEGF: vascular endothelial growth factor
WP: washed platelets
WT: wild type

## 1 Introduction

Myelin is an insulating material that wraps around axons, forming the myelin sheath. In the central nervous system (CNS), oligodendrocytes are responsible for producing myelin, whereas in the peripheral nervous system (PNS), Schwann cells do so. Myelin facilitates saltatory conduction, enabling the rapid and efficient transmission of electrical impulses along axons. However, the role of myelin extends beyond electrical conduction; it also supports axonal metabolism (Simons et al. 2024) and survival and contributes to motor skill learning (McKenzie et al. 2014).

Multiple sclerosis (MS) is a demyelinating autoimmune disease of the CNS that affects over 2.5 million people worldwide. MS represents a major cause of neurological disability in young adults (Ebers and Sadovnick 1993; Noseworthy et al. 2000). The etiology of MS is still unclear; however, a key component of the disease is an autoimmune response directed against CNS myelin. Symptoms of MS include blurred or double vision, weakness in the arms and legs, cognitive disruption, fatigue, and others. Unfortunately, current MS treatments have limited efficacy, are associated with significant adverse effects, and lack repair‐promoting activity (Compston and Coles 2008; Fox and Ransohoff 2004; Lassmann 2007, 2018). An ideal therapy for MS would target not only the autoimmune/inflammatory component of the disease but it should also enhance structural and functional myelin repair.

## 2 Remyelination and Its Failure in Multiple Sclerosis

Remyelination is an endogenous regenerative process that restores damaged myelin sheaths (R. J. Franklin and Ffrench‐Constant 2008; R. J. M. Franklin et al. 2024; Smith et al. 1979; R. H. Woodruff and Franklin 1999). Oligodendrocyte progenitor cells (OPCs) are the primary cellular source for myelin regeneration. These cells are widely distributed throughout the CNS, comprising 5% to 8% of the total glial cell population. The remyelination process can be divided into three phases: OPC activation, recruitment, and differentiation (Bruce et al. 2010; R. J. Franklin and Kotter 2008). Each of these steps is tightly regulated by extrinsic and intrinsic factors that function as either inhibitors or activators of remyelination (Rivera et al. 2010). In response to demyelination, astrocytes and microglia become activated and produce factors that aid in recruiting monocytes from blood vessels, which differentiate into macrophages. It is important to note that microglia and macrophages play a crucial role in clearing myelin debris, and any dysregulation in this process can result in remyelination failure (Ruckh et al. 2012). Activated astrocytes, microglia, and macrophages also secrete signals that promote the activation and proliferation of OPCs, inducing the expression of oligodendrogenic genes such as Olig2 and Nkx2.2 (Fancy et al. 2004; Levine and Reynolds 1999; Redwine and Armstrong 1998; Schonrock et al. 1998; Wilson et al. 2006). OPCs' recruitment is regulated by the interaction between cell surface molecules and the extracellular matrix (Larsen et al. 2003) and is promoted by factors such as platelet‐derived growth factor AA (PDGF‐AA) and fibroblast growth factor 2 (FGF‐2) (Murtie et al. 2005; Rachel H. Woodruff et al. 2004; Zhou et al. 2006). Ultimately, OPCs establish contact with exposed axons and differentiate into remyelinating oligodendrocytes (R. J. Franklin and Kotter 2008; R. J. M. Franklin et al. 2024).

While remyelination is a robust regenerative response to myelin damage, it largely fails in advanced MS stages. OPC recruitment and their differentiation into myelinating cells are impaired, resulting in incomplete remyelination and chronic neurological impairment (R. J. Franklin 2002; Williams et al. 2007). Most studies support a failure in OPC differentiation and maturation in MS. OPCs are typically found in demyelinated areas but fail to differentiate and remyelinate (Chang et al. 2002; Kuhlmann et al. 2008; Reynolds et al. 2002; Tiane et al. 2023; Wolswijk 1998). Besides cell‐intrinsic restrictions, changes in the regenerative niche are associated with OPC function alterations during MS. Chronic MS brains show microenvironmental changes that limit remyelination. The lack of proregenerative cues contributes to remyelination impairment in MS. Also, the presence of inhibitory signals leads to an antioligodendrogenic niche interfering with remyelination (Kuhlmann et al. 2008). Therefore, revealing new mechanistic insights for the understanding of myelin repair as well as the identification of cues that hamper remyelination in MS represents essential milestones for the development of regenerative therapies for the treatment of this disease.

## 3 Platelet Biology and Its Alterations in Multiple Sclerosis: Evidence for a Role in Remyelination

Platelets, also known as thrombocytes, are small, oval, circulating, anucleate cells that, upon endothelial damage, form the hemostatic plug to stop blood leakage (Semple et al. 2011). In addition to their ability to aggregate, platelets store a variety of bioactive molecules (such as growth factors, cytokines, RNAs, and microparticles) that, under specific circumstances, are secreted into the extracellular space and can target other cell types (Boven et al. 2006; Brill et al. 2005; Chen et al. 2012; Lohmann et al. 2012; Schallmoser and Strunk 2013; Semple et al. 2011; Warnke et al. 2013; Zhang et al. 2011). For example, beyond hemostasis, platelet and platelet‐derived molecules can regulate inflammation and modulate both innate and adaptive immune responses (Anitua et al. 2024; Nurden 2011; Semple et al. 2011). Platelet function is not limited to hemostasis and inflammation (immunity); they also exhibit tissue‐regenerative activities. Indeed, thrombocytes can directly contribute to neuroregeneration. Following CNS damage, platelet‐released molecules such as platelet‐derived growth factor (PDGF), basic fibroblast growth factor (bFGF), epidermal growth factor (EGF), hepatocyte growth factor (HGF), and vascular endothelial growth factor (VEGF) modulate angiogenesis, neurogenesis, neuroprotection, and nerve regeneration (Hayon et al. 2013; Z. Li et al. 2011; Nurden 2011). Furthermore, platelets contain molecules known to modulate the response of OPCs to demyelination and influence remyelination. In fact, we have demonstrated that platelet lysate enhances the survival of neural stem/progenitor cells (NSPCs), which are an eventual alternative cellular source for oligodendrocytes (Kazanis et al. 2015). Thus, these observations suggest that platelets may modulate OPC function and contribute to remyelination.

While the previous evidence supports the positive impact of platelets on OPC function and myelin repair, some research indicates that platelets may have a harmful effect in MS. It is noteworthy that platelets and platelet‐derived molecules have been identified in chronic non‐remyelinated lesions from MS patients (Han et al. 2008; Langer et al. 2012; Lock et al. 2002; Simon 2012; Steinman 2012). Moreover, circulating platelets in MS patients are in a hyper‐reactive state (Sheremata et al. 2008), displaying increased adhesiveness and correlating with clinical severity (Sanders et al. 1968). Furthermore, MS patients show increased numbers of blood‐resident platelet microparticles, which further increase after clinical exacerbations (Marcos‐Ramiro et al. 2014). In the experimental autoimmune encephalomyelitis (EAE), an animal model for autoimmune‐mediated demyelination as observed in MS, circulating platelets gradually rise from 0 to 7 days post‐induction (dpi), while platelet numbers within CNS peak at 14 dpi (Sonia D'Souza et al. 2018). When platelets were depleted before clinical onset, using an anti‐CD42b antibody, EAE severity decreased (Kocovski et al. 2019; Langer et al. 2012). These findings indicate that platelet abnormalities associated with MS, such as increased numbers, favor disease progression and suggest that they may be involved in remyelination failure during MS.

## 4 Bimodal Action of Platelets on OPC Differentiation and Remyelination

In a recently published pioneering study, we have explored the role of platelets in remyelination (Philp et al. 2024). We first assessed the presence of platelets during remyelination. We performed lysolecithin (LPC)‐induced demyelinating lesions in the spinal cord white matter of wild type (WT) mice. This model has been extensively used in this research field, as LPC induces rapid and efficient local demyelination primarily affecting oligodendrocytes, with the limitation that it may also impact some astrocytes. We observed that, already at 3 days post‐lesion (dpl), CD41+ platelet transiently aggregates localizing in proximity to OPCs, followed by a progressive decrease in their numbers until remyelination is complete at 21 dpl (Philp et al. 2024). To rule out the possibility that blood‐borne platelet recruitment and CNS aggregation resulted from vascular damage caused by the needle itself, we injected PBS (vehicle) containing DAPI into the spinal cord, which allowed us to localize the site of administration. Under these conditions, we found no signs of demyelination and minimal platelet aggregation. Thus, we confirmed that platelet recruitment was specific to demyelination.

**FIGURE 1:**
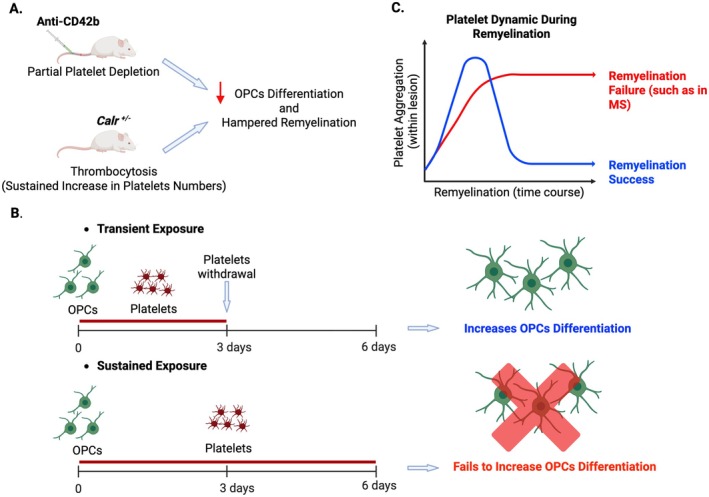
Effects of platelets on remyelination based on findings described in Philp et al. 2024. (A) The schema illustrates in vivo models demonstrating that both decreasing (partial platelet depletion induced by systemic administration of anti‐CD42b) and increasing (*Calr* ^*+/−*^ mutant) circulating platelets reduce oligodendrocyte progenitor cell (OPC) differentiation and hinder remyelination. (B) The schema depicts the In vitro effects of either transient or sustained exposure of OPCs to platelets. Note that transient exposure enhances OPC differentiation, whereas sustained exposure fails to do so. (C) The graph illustrates platelet aggregation within a demyelinated lesion throughout the course of remyelination. Note that while transient platelet aggregation occurs during a successful remyelination process, sustained aggregation (as it happens in MS) may lead to remyelination failure. Created with BioRender.com.

Next, we investigated whether circulating platelets modulate OPC function in vivo; we used a platelet depletion model that was previously described (Kocovski et al. 2019; Morodomi et al. 2020). Briefly, to induce partial depletion of circulating platelets, we administered anti‐CD42b antibody in WT mice at 3 dpl, and OPC function was evaluated during remyelination. We found that partial depletion of circulating platelets impaired OPC differentiation and remyelination (Philp et al. 2024). Interestingly, no signs of blood–brain barrier (BBB) alterations were observed, as platelet depletion did not lead to increased fibrinogen extravasation (Philp et al. 2024). Moreover, we found no changes in the number of IBA1‐expressing microglia/macrophages present in the lesion, nor in their polarization state or their capability to clear myelin debris, following platelet depletion (Philp et al. 2024). These findings collectively suggest that platelet depletion directly reduces OPC differentiation during remyelination, without affecting BBB stability or macrophage/microglia‐mediated neuroinflammation (Figure 1A).

Later, we set out to investigate how platelets might regulate OPC differentiation. Our initial in vivo observations showed a transient aggregation of platelets upon demyelination, prompting us to transiently expose OPCs to washed platelets (WP) In vitro and assess the generation of differentiated oligodendrocytes. We incubated OPCs in the presence of WP for 3 days, then evaluated OPC differentiation 3 days after WP withdrawal (transient exposure). Additionally, considering the presence of platelets in nonremyelinating MS lesions, we conducted a prolonged exposure of OPCs to WP for up to 6 days In vitro (sustained exposure). Surprisingly, we found that transient exposure to WP enhanced OPC differentiation, while sustained exposure to WP suppressed this effect (Philp et al. 2024) (Figure 1B). Furthermore, these results were replicated when OPCs were exposed to platelet lysate (transient or sustained), suggesting that molecules within platelets are responsible for bimodulating OPC differentiation.

Finally, in line with previous findings, we aimed to assess whether sustained exposure to platelets in vivo could negatively impact the generation of new remyelinating oligodendrocytes. To address this, we used a conditional mouse knock‐in model with a heterozygous mutation in the calreticulin gene (*Calr* ^*+/−*^), controlled by the Vav1 hematopoietic promoter, which results in persistent thrombocytosis (an increase in circulating platelet numbers) (J. Li et al. 2018). This study was restricted to the heterozygous condition, as homozygous mice (*Calr^−/−^*) also exhibit alterations in other hematopoietic cells, which represent a limitation of this animal model. We then induced a demyelinating lesion in the *Calr* ^*+/−*^ model and evaluated OPC function during remyelination. As expected, we observed a persistent increase in both circulating and lesion‐recruited platelets in *Calr* ^*+/−*^ thrombocytosis mice, indicating that this model is suitable for evaluating the in vivo consequences of sustained platelet exposure on OPC differentiation (Philp et al. 2024). Our observations revealed that *Calr* ^*+/−*^ mice exhibited a significant reduction in the number of new oligodendrocytes during remyelination compared to WT mice (Philp et al. 2024) (Figure 1A). Furthermore, we found a significant negative correlation between the number of circulating platelets and the number of differentiated oligodendrocytes (Philp et al. 2024). Therefore, sustained in vivo exposure to platelets, as seems to occur in chronic MS lesions, is detrimental to OPC differentiation during remyelination.

## 5 Final Remarks

Remyelination is a strong regenerative response to myelin damage, but it often fails in MS. Current treatments for MS are not very effective, can cause side effects, and do not focus on enhancing myelin repair. Identifying the molecular and cellular cues that regulate OPC function is crucial for understanding why remyelination fails in MS and for developing regenerative therapies.

Our recent study shows that circulating platelets play a beneficial role in remyelination; however, sustained abnormal platelet activity can be detrimental to myelin repair (Philp et al. 2024). Transient platelet aggregation occurs during a successful remyelination process, while sustained aggregation within lesions, as observed in MS, may lead to remyelination failure (Figure 1C). Our study indicates that platelets directly influence OPC function. While transient contact with circulating platelets supports OPC differentiation (beneficial role), prolonged remyelination (detrimental role) (Philp et al. 2024). Future directions aim to identify the underlying molecular and cellular mechanisms by which platelets exert their complex bimodal contribution to OPC differentiation. In light of this, considering the high content of PDGF molecules in platelets and the strong expression of their receptors by OPCs, the PDGF‐AA/PDGFRα pathway emerges as an attractive candidate for further investigation. Overall, all these findings together position platelets as significant contributors to remyelination failure in MS and highlight their potential as a target for developing regenerative strategies to treat the disease.

## Author Contributions

**Francisco J. Rivera:** conceptualization, funding acquisition, writing – original draft, writing – review and editing, project administration, supervision. **Amber R. Philp:** writing – review and editing, conceptualization. **Carolina R. Reyes:** writing – review and editing. **Carlos Valenzuela‐Krugmann:** writing – review and editing. **Maria Elena Silva:** visualization, writing – review and editing.

## Disclosure

The authors have nothing to report.

## Conflicts of Interest

The authors declare no conflicts of interest.

## Peer Review

The peer review history for this article is available at https://www.webofscience.com/api/gateway/wos/peer‐review/10.1111/jnc.70268.

## Data Availability

Data sharing is not applicable to this article because no new data were created or analyzed.
